# METTL14‐Mediated N6‐Methyladenosine Exacerbates Acute Lung Injury via USP30/NCOA4‐Dependent Ferroptosis

**DOI:** 10.1002/mco2.70959

**Published:** 2026-08-28

**Authors:** Aming Sang, Qiongyue Zhang, Xianwu Zhou, Xiaohua Wang, Jin Wang, Xuemin Song, Li Li, Ling Li, Xinyi Li

**Affiliations:** ^1^ Department of Anesthesiology Zhongnan Hospital of Wuhan University Wuhan Hubei China; ^2^ Hubei Provincial Engineering Research Center of Minimally Invasive Cardiovascular Surgery Wuhan Hubei China; ^3^ Department of Cardiovascular Surgery Zhongnan Hospital of Wuhan University Wuhan Hubei China; ^4^ Department of Critical Care Medicine Zhongnan Hospital of Wuhan University Wuhan China; ^5^ Clinical Research Center of Hubei Critical Care Medicine Wuhan Hubei China; ^6^ Zhongnan Hospital of Wuhan University, Institute of Hepatobiliary Diseases of Wuhan University, Transplant Center of Wuhan University, National Quality Control Center for Donated Organ Procurement, Hubei Key Laboratory of Medical Technology on Transplantation, Hubei Provincial Clinical Research Center for Natural Polymer Biological Liver Wuhan Hubei China

**Keywords:** acute lung injury, ferroptosis, ischemia‐reperfusion, N6‐methyladenosine, ubiquitination

## Abstract

Ischemia/reperfusion (I/R)‐induced acute lung injury (ALI) is a severe clinical syndrome characterized by alveolar epithelial damage, oxidative stress, and acute respiratory failure, but the epitranscriptomic mechanisms linking N6‐methyladenosine (m6A) modification to ferroptosis remain unclear. Clinical profiling of end‐stage acute respiratory distress syndrome (ARDS) patients showed hyperinflammation, reflected by elevated C‐reactive protein and procalcitonin, coagulopathy with abnormal D‐dimer levels, and severely impaired gas exchange. Reanalysis of a public murine single‐cell RNA sequencing dataset revealed enrichment of RNA methylation‐, ubiquitination‐, and ferroptosis‐related programs in injured lung epithelial cells. Integrated methylated RNA immunoprecipitation sequencing and RNA sequencing of I/R‐injured mouse lungs further identified methyltransferase‐like 14 (METTL14) and YTH N6‐methyladenosine RNA‐binding protein 1 (YTHDF1) as candidate m6A regulators. Human ARDS tissues and mouse I/R lungs confirmed increased METTL14, ubiquitin‐specific peptidase 30 (USP30), and nuclear receptor coactivator 4 (NCOA4) expression. Mechanistically, METTL14‐mediated m6A modification enhanced USP30 expression through YTHDF1 recognition, whereas USP30 stabilized NCOA4 by reducing K48‐linked polyubiquitination. Stabilized NCOA4 promoted ferritinophagy, Fe^2^^+^ accumulation, lipid peroxidation, and epithelial ferroptosis. These findings identify a METTL14/YTHDF1‐USP30‐NCOA4 axis as a potential therapeutic target for ferroptosis‐driven I/R‐induced ALI.

## Graphical abstract

METTL14‐mediated N6‐methyladenosine aggravates ischemia/reperfusion‐induced acute lung injury by enhancing YTHDF1‐dependent USP30 expression. USP30 stabilizes NCOA4 through deubiquitination, thereby promoting ferritinophagy, iron accumulation, lipid peroxidation, and ferroptosis in lung epithelial cells. Targeting this axis may provide a therapeutic strategy for ferroptosis‐driven lung injury.

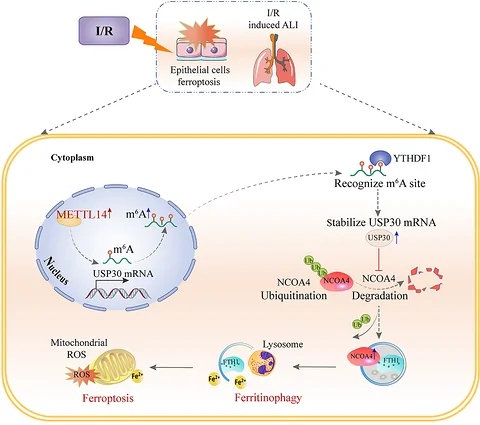

## 1 Introduction

Ischemia/reperfusion (I/R)‐induced acute lung injury (ALI), a critical clinical challenge, arises in diverse medical settings, including organ transplantation, general surgery, circulatory arrest, and cardiothoracic trauma [1], contributing substantially to global morbidity and mortality. In the lungs, I/R injury is a critical driver of ALI, primarily mediated by cellular damage triggered during reperfusion following ischemic hypoxia [2]. Ferroptosis, a newly identified form of programmed cell death, is characterized by iron‐dependent accumulation of reactive oxygen species (ROS) and lipid peroxidation [3]. Morphologically, ferroptotic cells exhibit mitochondrial condensation, reduced cristae, and increased membrane density [4]. Biochemically, ferroptosis is mainly characterized by glutathione depletion, decreased activity of glutathione peroxidase 4 (GPX4), and ROS overproduction [5, 6]. Mechanistically, cytoplasmic iron homeostasis is maintained under physiological conditions by storing iron within heavy‐chain ferritin (FTH1). FTH1, via its ferroxidase activity, catalyzes the conversion of Fe^2+^ to Fe^3+^, preventing harmful Fenton reactions between Fe^2+^ and hydrogen peroxide [7, 8]. However, under pathological stress such as inflammation or tissue injury, nuclear receptor coactivator 4 (NCOA4) functions as a cargo receptor for FTH1, directing it to lysosomes for degradation via encapsulation into autophagosomes [9]. This process, termed ferritinophagy, facilitates the lysosomal breakdown of FTH1, liberating Fe^2^^+^ into the cytoplasm [10]. The released Fe^2^^+^ subsequently accumulates in the mitochondrion, where it participates in the Fenton reaction, generating mitochondrial ROS and cellular lipid peroxides, which collectively trigger ferroptosis [11].

N6‐methyladenosine (m6A), the methylation of the N6 position of adenosine in RNA, represents the most common mRNA modification in mammals [12]. This epigenetic mechanism dynamically regulates mRNA expression prior to translation through three classes of enzymes: m6A‐binding proteins (readers), methyltransferases (writers), and demethylases (erasers), which collectively modulate RNA stability, degradation, transcription, and splicing [13]. The m6A modification process initiates in the nucleus where “writer” proteins, such as methyltransferase‐like 3 (METTL3) and methyltransferase‐like 14 (METTL14), catalyze the site‐specific RNA methylation [14]. Once the m6A‐modified mRNA is transported to the cytoplasm, it is recognized by reader proteins that determine its fate by either promoting translation or degradation. The “reader” protein, YTH N6‐methyladenosine RNA‐binding protein 1 (YTHDF1), stabilizes the mRNA by binding to the m6A site, thereby enhancing its expression and promoting protein translation [15]. YTHDF2, another “reader” protein, binds to the m6A site and promotes mRNA degradation [16]. Conversely, erasers like FTO and ALKBH5 reversibly remove the methyl group, resetting the m6A landscape [17, 18]. Notably, METTL3‐mediated m6A hypermethylation has been implicated in cardiomyocyte apoptosis during myocardial I/R injury [19, 20]. However, the dominant m6A writer involved in lung I/R injury remains unclear, and findings from myocardial I/R cannot be directly extrapolated to the lung. In our subsequent lung‐based analyses, METTL14 showed more consistent dysregulation, which prompted us to focus on METTL14 in the present study.

The ubiquitin‐proteasome system (UPS), a principal pathway for targeted protein degradation [21], involves sequential enzymatic actions of ubiquitin (Ub), E1 activating enzyme, E2 conjugating enzyme, E3 ligases, and deubiquitinases (DUBs) [22]. Among ∼100 human DUBs, the ubiquitin‐specific protease (USP) family is the most structurally and functionally characterized [23]. Ubiquitin‐specific peptidase 30 (USP30), a highly conserved member of the human DUBs/USPs family, is expressed in multiple organs, including the heart, liver, kidney, lung, and brain, with upregulated expression under stress conditions [24, 25]. Located on the outer mitochondrial membrane, USP30 counteracts ubiquitination events to suppress excessive mitophagy, thereby maintaining mitochondrial homeostasis [26]. This activity has been linked to various diseases, including cancer and Parkinson's disease [27]. Despite USP30's well‐established impact on mitochondrial function and disease development, its role in I/R injury remains enigmatic.

Building on emerging evidence and preliminary findings, we propose a mechanistic pathway wherein METTL14‐mediated m6A modification enhances USP30 mRNA stability by promoting YTHDF1 recognition of m6A motifs. This post‐transcriptional regulation elevates USP30 expression, thereby inhibiting NCOA4 ubiquitination and degradation, which ultimately leads to ferroptosis in lung epithelial cells during I/R injury. To investigate this hypothesis, we employed a multifaceted approach including high‐throughput RNA sequencing (RNA‐seq), m6A‐specific methylated RNA immunoprecipitation sequencing (MeRIP‐seq), and luciferase reporter assays. We further validated the clinical relevance of this pathway using human samples and preclinical models, aiming to elucidate the molecular mechanisms underlying METTL14‐mediated ferroptosis in I/R lung epithelial cells and identify novel therapeutic strategies for I/R‐induced ALI.

## 2 Results

### 2.1 Single‐Cell Transcriptomics Combined With m6A Epitranscriptomics Identifies Ferroptosis Pathways Associated With m6A Methylation Dynamics

**FIGURE 1:**
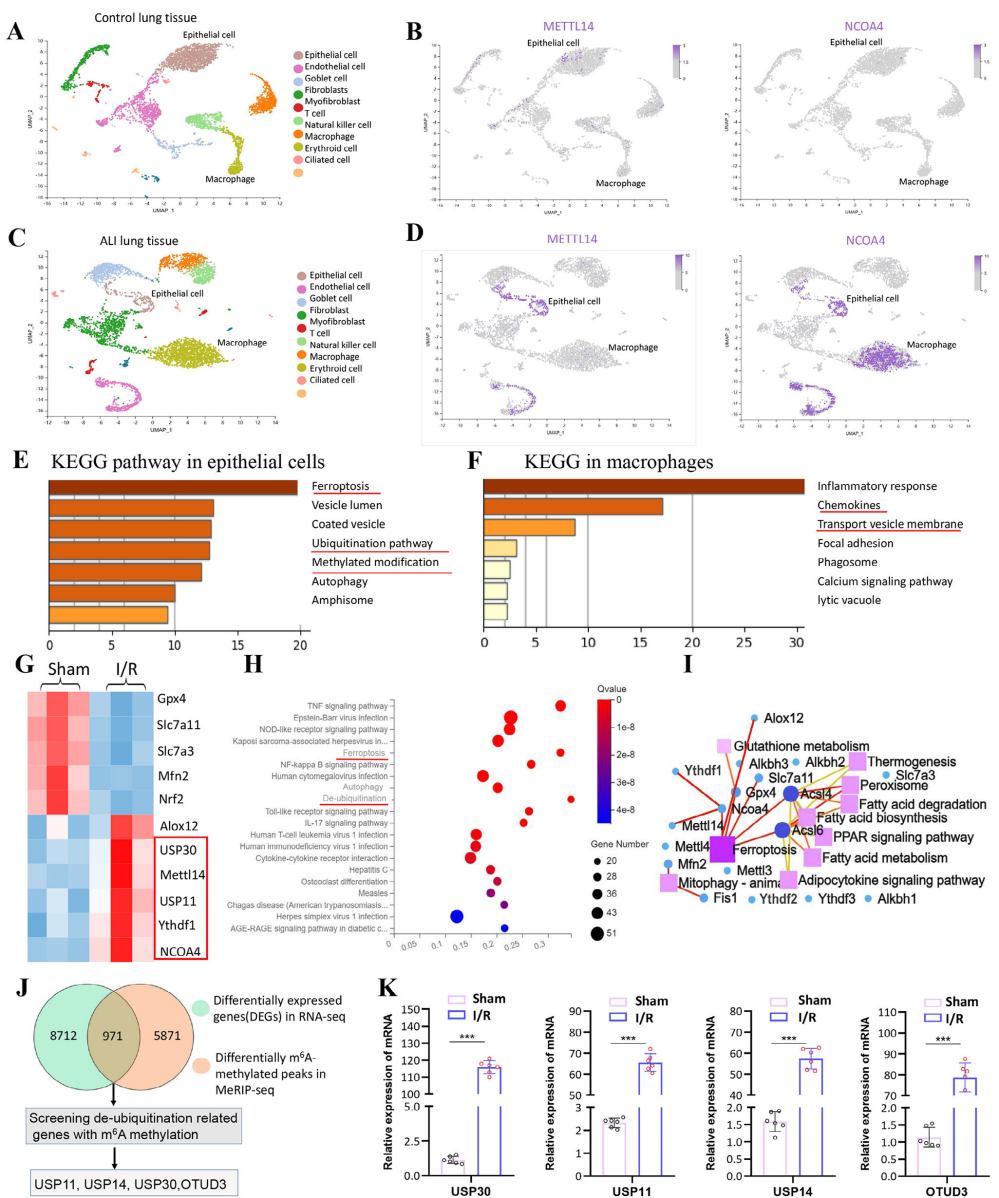
Reanalysis of a public murine scRNA‐seq dataset together with m6A epitranscriptomics identifies ferroptosis‐related pathways associated with m6A dynamics. (A, C) UMAP plots showing cell clusters in control and injured mouse lungs from GEO dataset GSE264278. (B, D) Feature plots of METTL14 and NCOA4 expression. (E, F) Pathway enrichment analysis in epithelial cells and macrophages, respectively. (G) Heatmap of differentially expressed genes in sham and I/R mouse lungs identified by RNA‐seq. (H) Differential m6A peak analysis and KEGG enrichment based on MeRIP‐seq. (I) Gene interaction network derived from enriched pathways. (J) Workflow for intersecting RNA‐seq and MeRIP‐seq results. (K) qRT‐PCR validation of candidate deubiquitinase genes in sham and I/R mouse lungs. For panels G–K, *n* = 6 mice per group. Data are shown as the mean ± SD. *p < 0.05, **p < 0.01,***p < 0.001 for indicated comparisons.

To establish the clinical relevance of severe lung injury in the human cohort, we first summarized the baseline characteristics and laboratory findings of patients with end‐stage ARDS. Clinical profiling showed a systemic inflammatory and coagulation‐disturbance profile, characterized by elevated C‐reactive protein and procalcitonin levels, abnormal D‐dimer levels, and severely impaired oxygenation indices (Table S1). These clinical features supported the presence of profound inflammatory injury and gas‐exchange dysfunction in the human lung specimens used for validation. We next reanalyzed a publicly available murine single‐cell RNA sequencing (scRNA‐seq) dataset from the Gene Expression Omnibus (GEO; GSE264278). UMAP analysis revealed altered epithelial cell distributions and increased expression of METTL14 and NCOA4 in injured lungs relative to controls (Figure 1A–D). Differential expression and pathway enrichment analyses further identified methylation‐, ubiquitination‐, and ferroptosis‐related pathways within the epithelial compartment (Figure 1E,F). To further identify m6A methylation‐related genes in I/R‐induced ALI, we performed RNA‐seq and MeRIP‐seq on lung tissues from I/R and sham‐operated mice. RNA‐seq demonstrated enrichment of ferroptosis‐ and ubiquitination‐related pathways in I/R‐induced ALI (Figure 1G–I). MeRIP‐seq further identified genes with significant m6A modification. Focusing on DUB family members, intersection analysis of RNA‐seq and MeRIP‐seq data identified a subset of ubiquitination‐related genes with altered m6A modification in I/R‐induced ALI (Figure 1J,K). Quantitative reverse transcription polymerase chain reaction (qRT‐PCR) analysis showed increased expression of USP11, USP14, USP30, and OTUD3 mRNA in the lungs of mice with ALI, among which USP30 showed the most pronounced increase.

### 2.2 Significant Increase in m6A Methylation Levels in Lung Tissue of I/R ALI Mice

**FIGURE 2:**
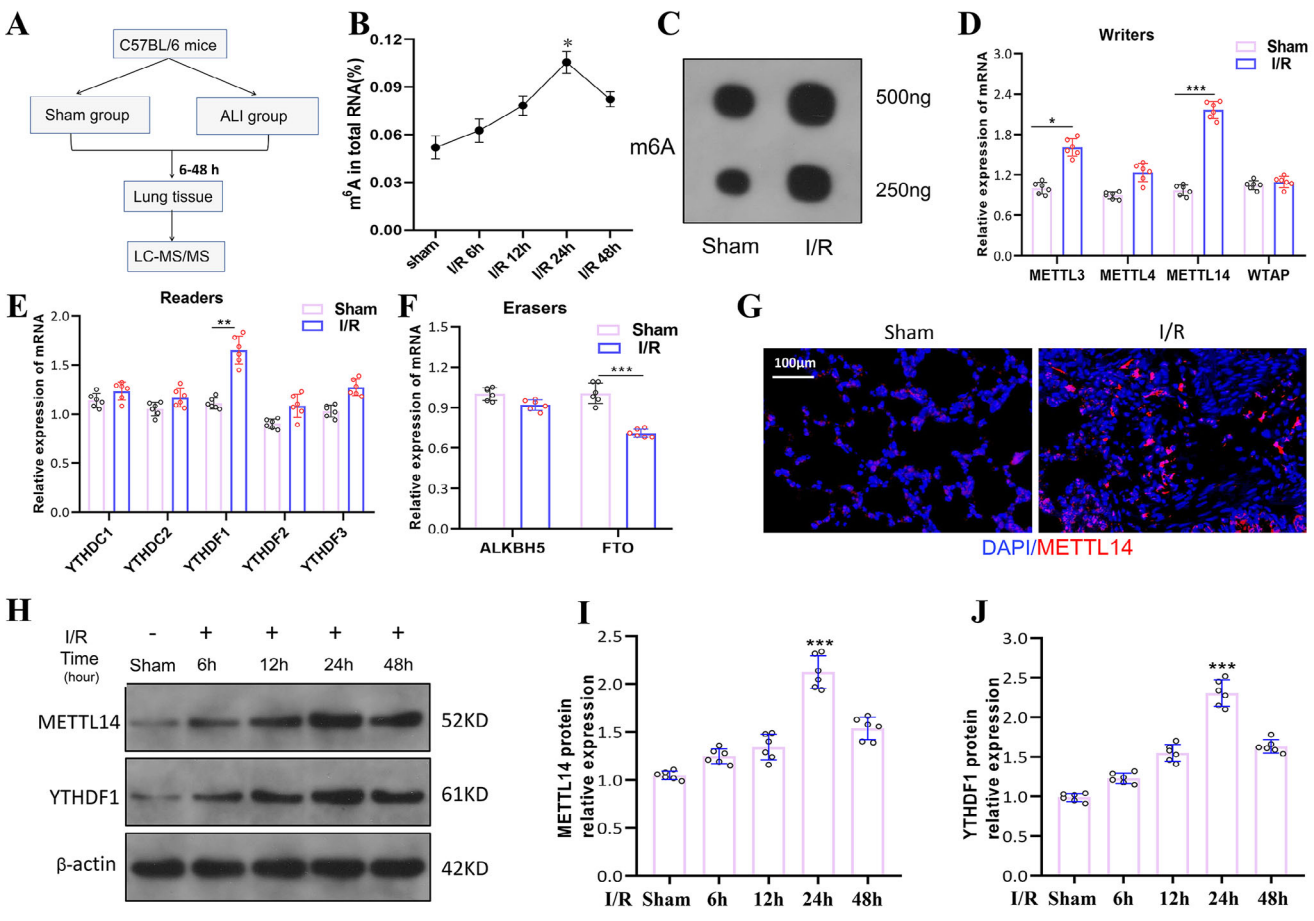
I/R‐induced ALI in mice is associated with increased m6A methylation in lung tissue. (A) Tissue collection flowchart. (B) LC‐MS/MS‐based determination of total m6A content in lung tissue. (C) Dot blot assay for m6A levels in lung tissue. (D–F) qPCR‐based quantification of m6A regulator gene expression. (G) Immunofluorescence staining of METTL14 in lung tissue. (H–J) Quantification of METTL14 and YTHDF1 protein levels in lung tissue using western blot at different time points following I/R model establishment. (*n* = 6). Data are shown as the mean ± SD. *p < 0.05, **p < 0.01,***p < 0.001 for indicated comparisons.

To investigate m6A methylation dynamics in I/R‐induced ALI, we established a murine I/R lung injury model [28]. Global m6A levels in lung tissues were quantified at 6‐, 12‐, 24‐, and 48‐h post‐I/R surgery (sham‐operated group as the control). LC‐MS/MS analysis revealed a significant increase in m6A methylation at 24‐h post‐I/R (*p* < 0.01 vs. sham; Figure 2A,B), which prompted the selection of this time point for subsequent experiments. Dot blot experiment corroborated these findings (Figure 2C). qRT‐PCR analysis of m6A methylation‐regulatory genes demonstrated upregulated METTL3, METTL14 (Writer), and YTHDF1 (Reader) alongside downregulated FTO (Eraser) in the I/R groups compared to the sham groups (Figure 2D–F). Immunofluorescence (IF) staining confirmed METTL14 overexpression in I/R lungs (Figure 2G), while western blot revealed peak METTL14 and YTHDF1 protein expression at 24‐h post‐I/R (Figure 2H–J). Together, these findings highlight m6A hyper‐methylation as a hallmark of I/R‐induced lung injury, driven by METTL14/YTHDF1 upregulation and FTO suppression.

### 2.3 METTL14 Deficiency in Lung Epithelial Cells Attenuates Ferroptosis and I/R Injury in the Lung

**FIGURE 3:**
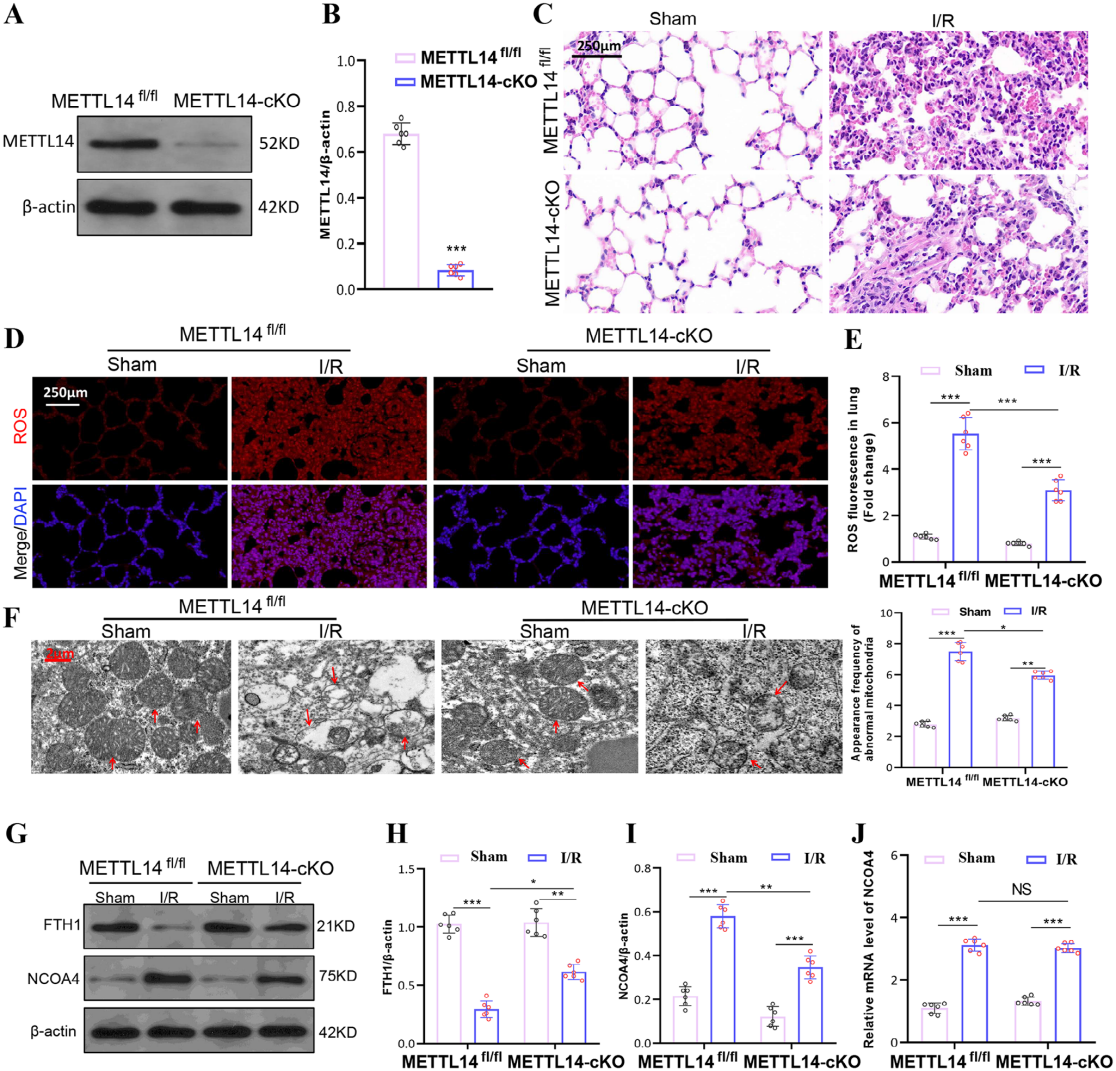
METTL14 deficiency in lung epithelial cells attenuates ferroptosis and I/R injury in the lung. (A and B) Validation of METTL14 conditional knockout in mice lung epithelial cells. (C) HE staining of lung tissue. (D and E) ROS fluorescence staining of lung tissue and quantification of fluorescence intensity. (F) TEM images demonstrate alterations in mitochondria (arrows indicate mitochondria) in type II epithelial cells of lung tissue. (G–I) Quantification of FTH1 and NCOA4 protein levels in lung tissue by western blot. (J) qPCR for measuring NCOA4 mRNA levels (*n* = 6). Data are shown as the mean ± SD. *p < 0.05, **p < 0.01,***p < 0.001 for indicated comparisons.

To elucidate the role of METTL14 in I/R‐induced ALI, we generated lung epithelial‐specific METTL14 knockout mice (METTL14‐cKO). Western blot analysis confirmed efficient METTL14 ablation in METTL14‐cKO mice (Figure 3A,B). Following I/R treatment, METTL14^fl/fl^ mice exhibited severe lung pathology, including alveolar hemorrhage and extensive inflammatory cell infiltration, which were significantly attenuated in METTL14‐cKO mice (Figure 3C). Similarly, ROS fluorescence intensity, elevated in METTL14^fl/fl^ I/R lungs, was suppressed in METTL14‐cKO mice (Figure 3D,E). We performed 4‐HNE and TUNEL staining in our experiments (Figure S2A–D). At the examined time point, TUNEL staining did not show a significant difference between the METTL14^fl/fl^+I/R and METTL14‐cKO+I/R groups, suggesting that METTL14 deficiency did not produce a detectable effect on epithelial apoptosis under these conditions. However, following I/R treatment, METTL14^fl/fl^ mice exhibited a significant increase in 4‐HNE IF intensity, and this oxidative stress response was markedly attenuated in METTL14‐cKO mice. Furthermore, transmission electron microscopy (TEM) revealed mitochondrial cristae loss and vacuolization, and phenotypes ameliorated in METTL14‐cKO mice (Figure 3F). Mechanistically, METTL14 deficiency increased FTH1 and decreased NCOA4 protein levels in I/R lungs (Figure 3G–I), suggesting suppression of ferroptosis in the lung tissue of mice subjected to I/R injury. Notably, NCOA4 and FTH1 mRNA levels were not significantly altered in the METTL14‐cKO‐ALI group relative to the METTL14^fl/fl^‐ALI group (Figure 3J, Figure S2E), despite clear changes at the protein level. This discrepancy suggested that METTL14 was unlikely to regulate NCOA4 primarily at the transcriptional level and prompted us to examine post‐translational control of NCOA4 stability. Consistent with this interpretation, loss of METTL14 accelerated NCOA4 degradation, supporting a role for ubiquitin‐proteasome‐dependent regulation (Figure S1F,G). We therefore focused on ubiquitination‐related mechanisms that could account for the observed protein‐level changes.

### 2.4 USP30 Interacts With NCOA4 and Mediates NCOA4 Protein Stabilization

**FIGURE 4:**
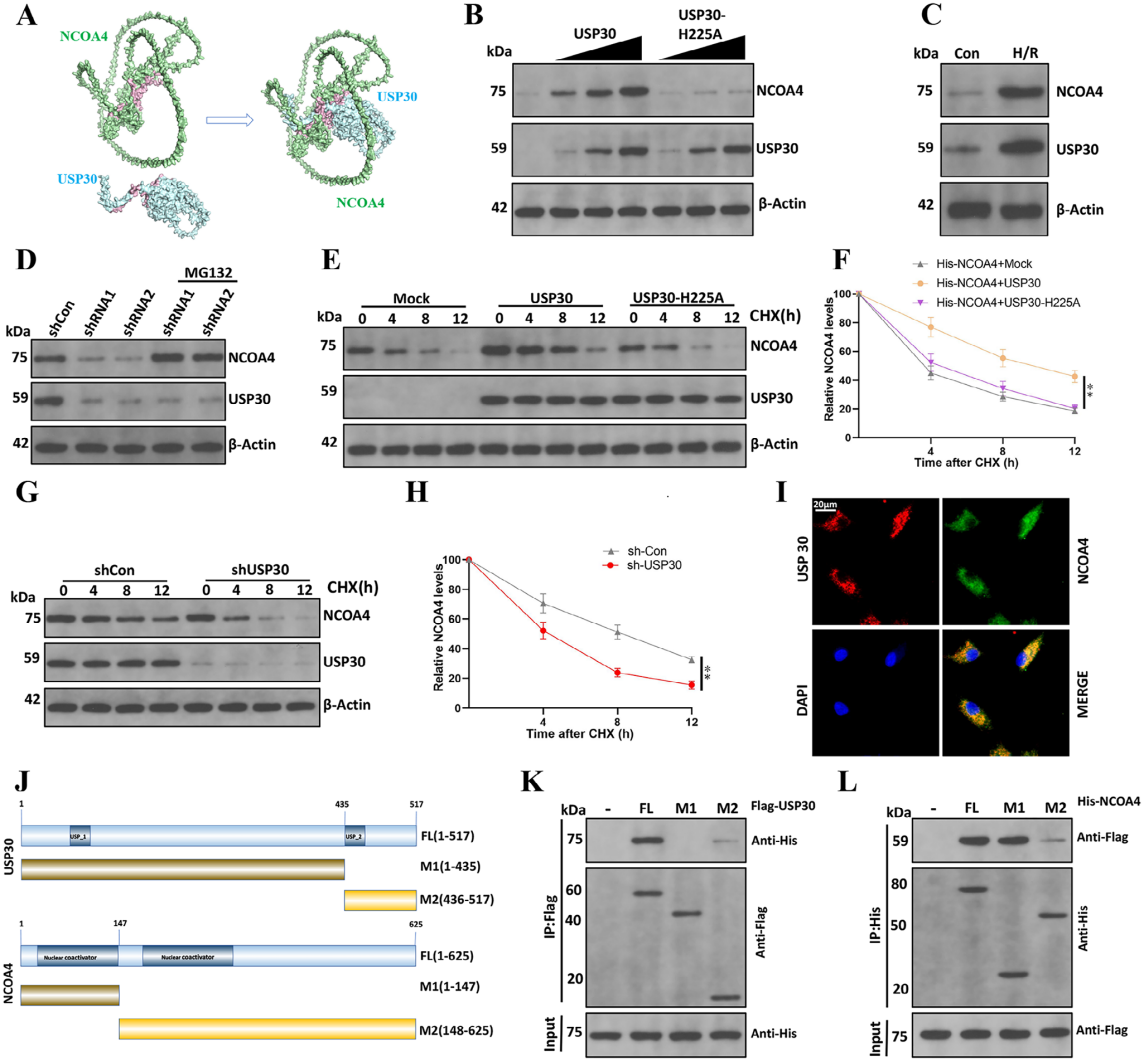
USP30 interacts with NCOA4 and mediates NCOA4 protein stabilization. (A) The three‐dimensional structure of NCOA4 (UniProt ID:Q13772) bound to USP30 (UniProt ID:Q70CQ3) was predicted using AlphaFold3. (B) TC‐1 cells were transfected with either wild‐type USP30 or USP30‐H225A, and cell lysates were analyzed by western blotting using an anti‐NCOA4 antibody. (C) Quantification of NCOA4 and USP30 protein expression in the H/R group by western blot. (D) Two separate USP30 shRNAs were transfected into TC‐1 cells, and after 8 h of treatment with or without the proteasome inhibitor MG‐132 (20 µM), USP30 and NCOA4 were measured. (E and F) TC‐1 cells were treated with CHX (40 µg/mL), co‐transfected with NCOA4 and either wild‐type USP30 or USP30‐H225A, and then subjected to western blotting at different intervals. The levels of NCOA4 were measured in relation to β‐actin. (G and H) After treatment with CHX (40 µg/mL), TC‐1 cells stably expressing shRNA‐USP30 or control shRNA treatment were analyzed by western blotting at different time points (under H/R stress). (I) USP30 (red) and NCOA4 (green) were highly expressed in TC‐1 cells, as shown by confocal imaging. DAPI (blue) was used as a counterstain for the nuclei. (J) His‐tagged NCOA4, full‐length (FL) Flag‐tagged USP30, and multiple truncated fragments are shown schematically. (K) His‐NCOA4 and FL Flag‐tagged USP30 or truncated fragments were co‐transfected into TC‐1 cells. Flag beads were used for IP analysis on cell lysates, while His and Flag antibodies were used for Western blotting. (L) Flag‐USP30 and FL His‐tagged NCOA4 or truncated portions were co‐transfected into TC‐1 cells. HIS beads were used for IP analysis on cell lysates, and His and Flag antibodies were used for western blotting (*n* = 6). Data are shown as the mean ± SD. *p < 0.05, **p < 0.01,***p < 0.001 for indicated comparisons.

To investigate the potential interaction between USP30 and NCOA4, we predicted the three‐dimensional structure of their binding using AlphaFold3 (Figure 4A; the interaction between the two proteins is depicted as surface models, with interfacial regions colored in pink; Figure S3A–C). Transfection of TC‐1 cells with WT USP30, but not the catalytically inactive H225A mutant, increased NCOA4 protein levels in a dose‐dependent manner (Figure 4B), indicating that USP30 regulates NCOA4 through its DUB activity. Notably, we observed elevated expression levels of USP30 and NCOA4 in the I/R group (Figure 4C). Furthermore, USP30 deficiency led to a reduction in NCOA4 expression, an effect reversed by MG‐132, a proteasome inhibitor (Figure 4D). This suggests that USP30 deficiency promotes the degradation of NCOA4. To validate this conclusion, we conducted a CHX pulse‐chase experiment. The results showed that the overexpression of USP30, but not the H225A mutant, significantly delayed NCOA4 degradation (Figure 4E–F). Conversely, the knockdown of USP30 accelerated the degradation of NCOA4 (Figure 4G,H). Collectively, these findings indicate that USP30 regulates the stability of NCOA4. Confocal imaging revealed colocation of USP30 (red) and NCOA4 (green) in the cytoplasm (Figure 4I). Domain‐mapping experiments using truncated constructs localized the interaction to the M1 region of NCOA4 (amino acids 1–147) and the carboxyl‐terminal region of USP30 (amino acids 436–517) (Figure 4J–L).

### 2.5 USP30 Regulates the Deubiquitination of NCOA4

**FIGURE 5:**
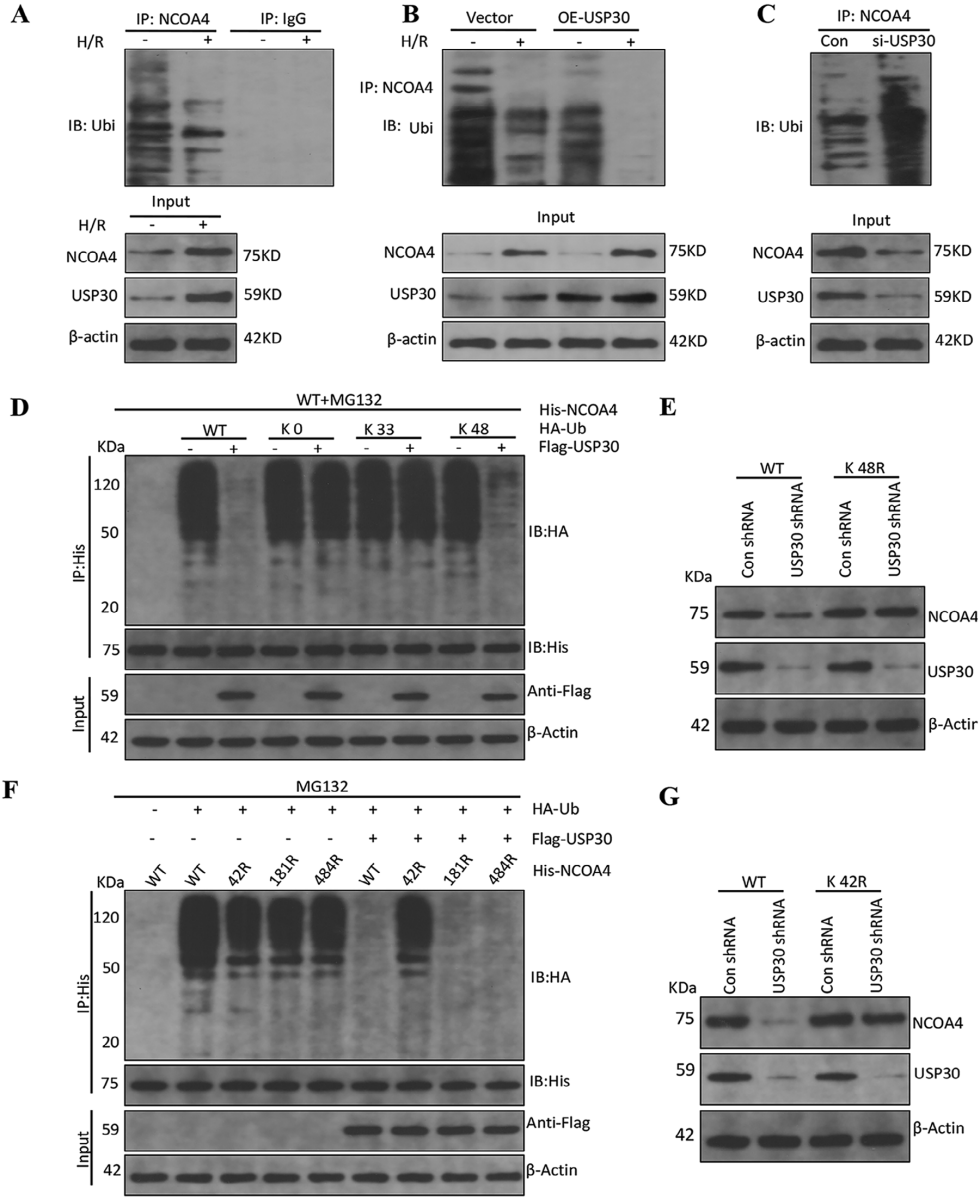
USP30 regulates the deubiquitination of NCOA4. (A–C) Co‐IP analysis revealed the protein expression levels of NCOA4 and its ubiquitination status under H/R conditions following USP30 overexpression or silencing. (D) TC‐1 cells were co‐transfected with His‐NCOA4, Flag‐USP30, and HA‐tagged ubiquitin constructs (Lys0, Lys33‐only, or Lys48‐only) for analysis of NCOA4 ubiquitination chains. (E) TC‐1 cells were transfected with either wild‐type Ub or Ub‐Lys48R and cultivated for 72 h in the presence of either control shRNA or USP30 shRNA. Western blotting with anti‐NCOA4 and anti‐USP30 antibodies was used to analyze cell lysates. (F) A control vector, Flag‐USP30, HA‐Ub, and either wild‐type His‐NCOA4 or K‐to‐R mutations were used to transfect TC‐1 cells. MG‐132 was used to treat the cells. Anti‐Flag beads were used to denature the samples, and then anti‐HA or anti‐Flag antibodies were used for immunoblotting. (G) Following transfection with either wild‐type Ub, wild‐type His‐NCOA4, or His‐NCOA4‐K42R, TC‐1 cells were grown for 72 h with either USP30 shRNA or control shRNA present. Western blotting with anti‐NCOA4 and anti‐USP30 antibodies was used to examine cell lysates (*n* = 6). Data are shown as the mean ± SD. *p < 0.05, **p < 0.01,***p < 0.001 for indicated comparisons.

To further explore the potential interaction between USP30 and NCOA4, we performed co‐immunoprecipitation (Co‐IP) assays. The findings revealed that under H/R conditions, USP30 overexpression suppressed NCOA4 ubiquitination and enhanced its stability, whereas silencing USP30 promoted ubiquitination and degradation (Figure 5A–C). To identify the ubiquitin linkage type targeted by USP30, we utilized a set of ubiquitin mutants retaining single lysine residues, while the others were substituted with arginine residues. USP30 specifically reduced K48‐linked (but not K33‐linked) polyubiquitination of NCOA4 (Figure 5D). Consistent with this, USP30 deficiency reduced NCOA4 levels, an effect rescued by expressing K48R ubiquitin (Figure 5E). Using phosphoSitePlus (https://www.phosphosite.org), we generated three lysine‐to‐arginine substitution mutants of NCOA4 (NCOA4‐K42R, ‐K181R, and ‐K484R). USP30 overexpression selectively reduced ubiquitination of the NCOA4‐K42R mutant (Figure 5F). In addition, the downregulation of NCOA4 expression due to USP30 deficiency was attenuated by the K42R mutation (Figure 5G). Together, these findings demonstrate that USP30 stabilizes NCOA4 by eliminating K48‐linked polyubiquitin chains at lysine 42.

### 2.6 METTL14 Promotes USP30 Expression by Mediating m6A Modification of USP30 mRNA

**FIGURE 6:**
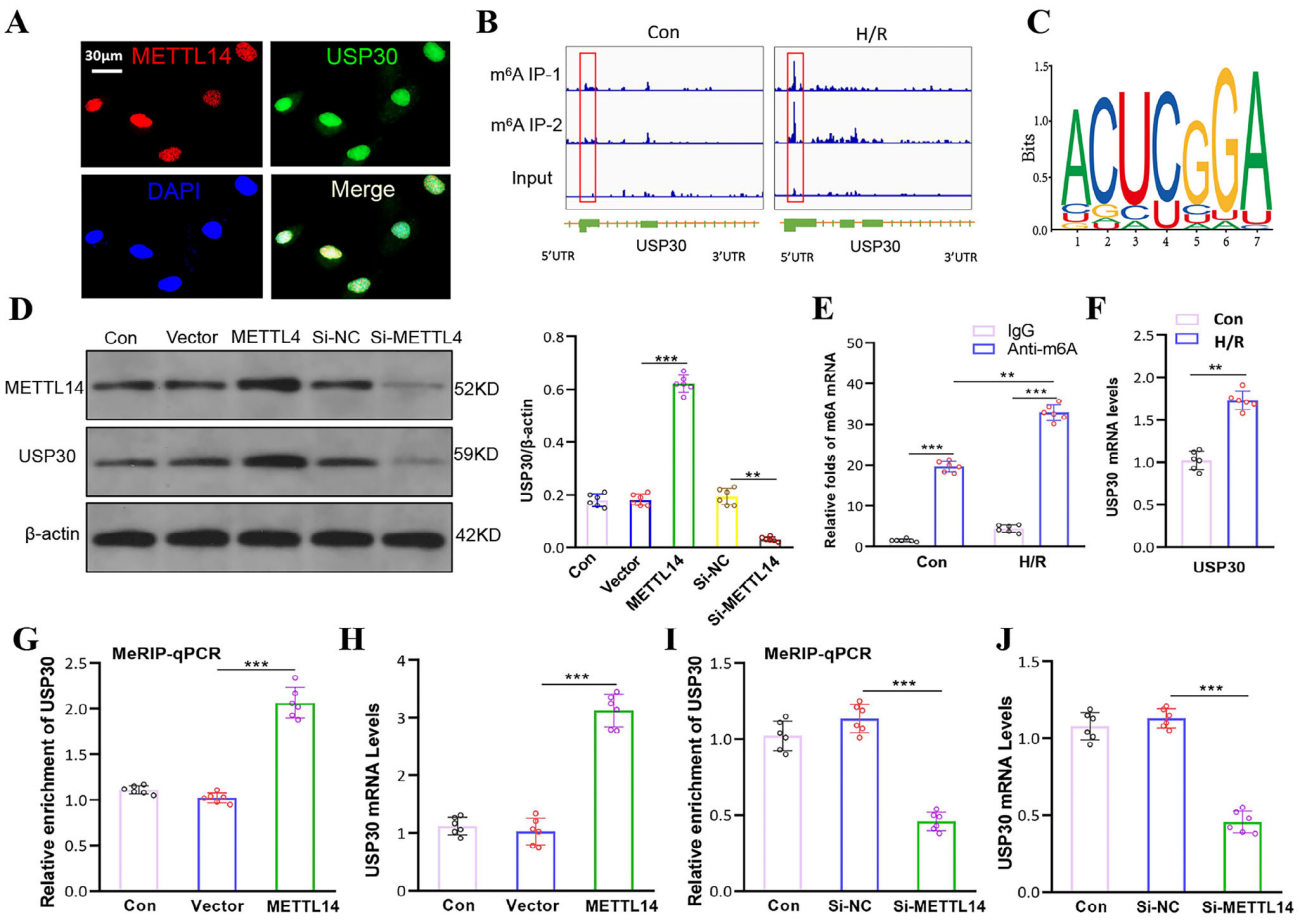
METTL14 promotes USP30 expression through m6A modification of USP30 mRNA. (A) Combined IF–RNA FISH assay showing METTL14 protein and USP30 transcript localization in TC‐1 cells. (B) MeRIP‐seq tracks showing m6A enrichment in the USP30 5′UTR under control and H/R conditions. (C) Enriched sequence motif identified from m6A peaks. (D) Immunoblot analysis of METTL14 and USP30 after METTL14 overexpression or silencing. (E and F) USP30 m6A abundance and USP30 mRNA levels in control and H/R‐treated cells. (G–J) USP30 m6A abundance and USP30 mRNA levels after METTL14 overexpression or knockdown. *n* = 6. Data are shown as mean ± SD. *p < 0.05, **p < 0.01, ***p < 0.001.

To further explore the molecular basis of METTL14‐mediated regulation of USP30, we performed a combined IF‐RNA FISH assay to visualize METTL14 protein and USP30 transcript localization in TC‐1 cells. This assay showed overlapping nuclear signals for METTL14 and USP30 (Figure 6A). Subsequent MeRIP coupled with m6A immunoprecipitation‐sequencing identified prominent m6A modification clusters in the 5'UTR region of USP30 mRNA (Figure 6B). Motif analysis further revealed significant enrichment of the ACUCG motif within m6A‐modified sites (Figure 6C). Functional validation demonstrated that METTL14 overexpression upregulated USP30 protein levels, whereas METTL14 silencing reduced them, establishing a dose‐dependent relationship (Figure 6D). The H/R treated cells exhibited elevated m6A levels and increased USP30 mRNA expression compared to control (Figure 6E,F). Concurrently, MeRIP‐qPCR and qPCR confirmed that METTL14 overexpression amplified both USP30 m6A modification and USP30 mRNA levels, while METTL14 silencing suppressed these effects (Figure 6G–J). These findings suggest that METTL14 enhances USP30 m6A modification and promotes USP30 protein expression.

### 2.7 YTHDF1 Recognizes the m6A‐Modified USP30 Transcript and Is Associated With Increased USP30 mRNA Stability

**FIGURE 7:**
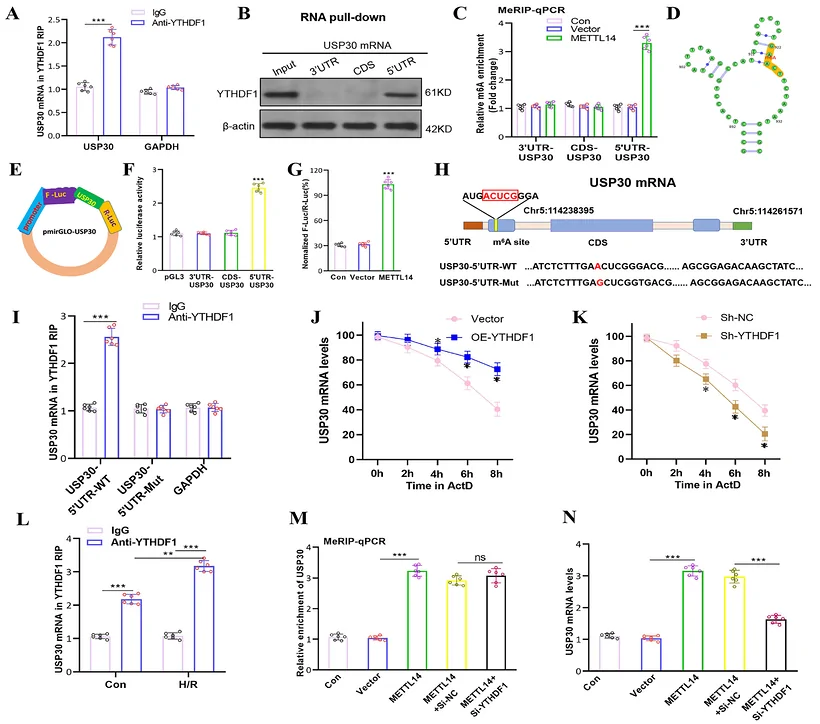
YTHDF1 recognizes the m6A‐modified USP30 transcript and is associated with increased USP30 mRNA stability. (A) RIP analysis of YTHDF1 binding to USP30 mRNA. (B) RNA pull‐down analysis showing the interaction region within the USP30 5′UTR. (C) MeRIP‐qPCR analysis of m6A abundance across USP30 transcript regions. (D) Predicted m6A motif within the USP30 5′UTR. (E‐G) Luciferase reporter assays evaluating the effect of METTL14 on USP30 5′UTR activity. (H) Schematic of the wild‐type and mutant USP30 5′UTR constructs. (I) RIP analysis of YTHDF1 binding to wild‐type and mutant USP30 transcripts. (J–K) RNA stability analysis after YTHDF1 overexpression or knockdown. (L) RIP analysis of YTHDF1–USP30 interaction under H/R conditions. (M and N) MeRIP‐qPCR and qPCR analyses after METTL14 overexpression and YTHDF1 silencing (*n* = 6). Data are shown as the mean ± SD. *p < 0.05, **p < 0.01,***p < 0.001 for indicated comparisons.

To determine whether the METTL14‐dependent regulation of USP30 involved the m6A reader protein YTHDF1, we conducted a series of experiments to investigate whether YTHDF1 can bind to USP30 mRNA and stabilize its mRNA levels. Moreover, the binding site was located in the 5'UTR region of USP30. RIP analysis demonstrated the significant enrichment of USP30 mRNA in the YTHDF1 samples compared to GAPDH, confirming direct interaction (Figure 7A). RNA pulldown localized this interaction to the 5'UTR of USP30 (Figure 7B). Concurrently, MeRIP‐qPCR validated a notable elevation in m6A methylation levels within the 5'UTR of USP30 following METTL14 overexpression (Figure 7C), consistent with METTL14's role in site‐specific m6A deposition, which is then recognized and bound to by YTHDF1. To further test this hypothesis, we identified a candidate m6A motif in the USP30 5′UTR (Figure 7D). Luciferase reporter assay utilizing pmirGLO‐USP30 construct demonstrated that overexpression of METTL14 significantly enhanced the translation efficiency in TC‐1 cells, implicating the 5'UTR as a functional METTL14 target (Figure 7E–G). To validate this, we constructed USP30 5'UTR‐wt and USP30 5'UTR‐mut construct (Figure 7H). RNP IP experiments revealed that USP30 5'UTR‐Mut markedly disrupted the binding of YTHDF1 to USP30 mRNA, while USP30 5'UTR‐WT remained unaffected (Figure 7I). RNA stability assays demonstrated that overexpression of YTHDF1 prolonged the half‐life of USP30 mRNA, whereas YTHDF1 deficiency shortened it (Figure 7J,K). Furthermore, the H/R treatment amplified the interaction between YTHDF1 and USP30 (Figure 7L). TC‐1 cells were divided into five groups (con, vector, METTL14, METTL14+si‐NC, and METTL14+si‐YTHDF1) to examine the interaction between YTHDF1 and METTL14 with USP30. Compared to the vector group, METTL14 overexpression significantly enhanced the m6A methylation of USP30. There was no significant difference between the METTL14+si‐NC and METTL14+si‐YTHDF1 groups, suggesting that the interaction of YTHDF1 with USP30 occurs after USP30 methylation (Figure 7M). Additionally, USP30 mRNA levels were significantly reduced in the METTL14+si‐YTHDF1 group compared to the METTL14+si‐NC group (Figure 7N). Taken together, these findings suggest that, in our experimental setting, YTHDF1 promotes USP30 expression at least in part by increasing USP30 mRNA stability and functions downstream of METTL14.

### 2.8 METTL14 Deficiency Attenuates Ferroptosis in Lung Epithelial Cells

**FIGURE 8:**
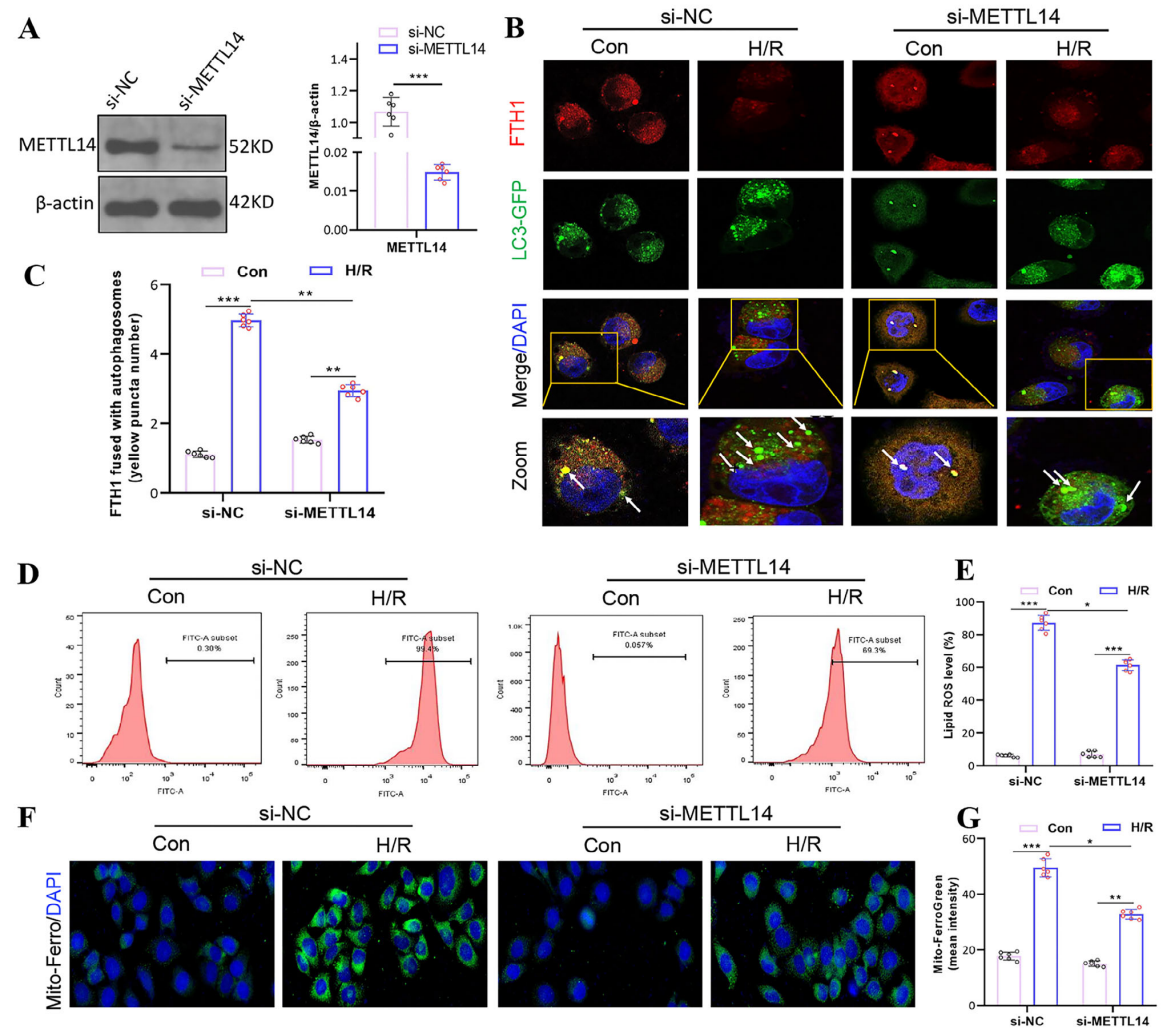
METTL14 deficiency attenuates ferroptosis in lung epithelial cells. (A) Immunoblot confirming METTL14 knockdown. (B) Representative confocal images showing LC3‐GFP and FTH1 colocalization. (C) Quantification of FTH1 fusion with autophagosomes. (D and E) Flow cytometry analysis of intracellular Lipid ROS levels. (F and G) Quantification of mitochondrial Fe^2+^ fluorescence intensity using Mito‐FerroGreen (*n* = 6). Data are shown as the mean ± SD. *p < 0.05, **p < 0.01,***p < 0.001 for indicated comparisons.

To investigate METTL14's role in regulating ferroptosis, we silenced METTL14 in TC‐1 murine lung epithelial cells using siRNA (si‐METTL14), as confirmed by western blot (Figure 8A), and exposed the cells to H/R. Subsequently, we transfected cells with LC3‐GFP autophagosome plasmids and used IF and confocal microscopy to assess FTH1 fusion with autophagosomes (a process termed ferritinophagy). The results showed that silencing METTL14 inhibited the autophagic degradation of FTH1, thereby attenuating cellular ferritinophagy and ferroptosis (Figure 8B,C). Flow cytometry revealed that H/R significantly enhanced lipid ROS production, while METTL14 deficiency mitigated these alterations (Figure 8D,E). Compared with the Con group, the H/R group exhibited a significant increase in ROS accumulation; however, both Fer‐1 treatment and METTL14 deficiency effectively attenuated this oxidative stress response, suggesting that METTL14 deficiency had a similar anti‐ferroptosis effect comparable to Fer‐1 (Figure S4A,B). Similarly, H/R‐induced mitochondrial Fe^2^^+^ accumulation was reduced in METTL14‐deficient cells (Figure 8F,G). These results identify METTL14 as a critical mediator of ferroptosis in lung epithelial cells under H/R stress.

## 3 Discussion

I/R injury disrupts the metabolic equilibrium of the ischemic organ, leading to pronounced tissue hypoxia and impaired microvascular function. Subsequent reperfusion exacerbates the activation of innate and adaptive immune responses, as well as the initiation of cell death pathways [1, 29]. While regulated cell death (RCD) modalities, including ferroptosis, necroptosis, and autophagy, have been implicated in lung I/R injury [30, 31], the precise mechanisms underlying these processes remain poorly defined. Clinically, I/R‐associated lung injury presents a considerable therapeutic challenge, frequently complicating surgical procedures like lung transplantation and cardiopulmonary bypass surgery. During ischemia, oxygen and nutrient deprivation disrupt cellular metabolism, resulting in cellular dysfunction and injury [30]. Paradoxically, reperfusion exacerbates tissue damage by triggering the production of ROS, provoking inflammatory responses, and compromising the integrity of endothelial and epithelial barriers [32]. Consequently, I/R lung injury significantly contributes to morbidity and mortality in clinical settings, highlighting the critical need for a comprehensive understanding of its underlying mechanisms to develop effective therapeutic strategies.

In this study, we examined how m6A‐related regulation interfaces with ferroptosis in I/R‐induced lung injury. Reanalysis of a public murine scRNA‐seq dataset showed epithelial enrichment of methylation‐, ubiquitination‐, and ferroptosis‐related pathways, and validation in human end‐stage ARDS tissues showed increased METTL14, USP30, and NCOA4 expression. In parallel, epithelial METTL14 deletion attenuated lung injury and ferroptosis in the murine I/R model. Taken together with the biochemical data, these results support a model in which METTL14/YTHDF1‐dependent regulation of USP30 contributes to NCOA4 stabilization and ferritinophagy‐associated ferroptosis.

m6A methylation is a dynamic epigenetic regulator critical for diverse biological processes, including cell cycle regulation, embryonic development, metabolic modulation, cellular differentiation, and the initiation and progression of diseases [14, 33, 34]. In this study, reanalysis of a public murine scRNA‐seq dataset revealed epithelial enrichment of pathways related to ferroptosis, methylation, and ubiquitination. Validation by immunohistochemical and biochemical analyses further supported coordinated changes in these pathways in severe lung injury.

Ubiquitination is a post‐translational modification that attaches ubiquitin to target proteins [35] and typically marks them for proteasomal degradation. Deubiquitination, mediated by DUBs, removes ubiquitin, preventing degradation and preserving free ubiquitin. DUBs play essential roles in diverse cellular processes, including apoptosis, autophagy, and immune responses [36]. Aberrant DUB activity has been implicated in various disease states, such as Alzheimer's disease and cancer, highlighting their potential as therapeutic targets [37]. Previous research demonstrated that METTL14 contributes to ALI by stabilizing NLRP3 expression [38]. Given the concurrent expression of METTL14 and NCOA4 in ALI, it is hypothesized that METTL14 may similarly stabilize NCOA4, thereby exacerbating ferroptosis and contributing to ALI pathogenesis. In parallel, USP30 is a critical DUB that plays a crucial role in regulating protein stability, signal transduction, and cellular processes [39]. It was predicted to interact with NCOA4 via structural modeling (AlphaFold3). Transfection of TC‐1 cells with wild‐type USP30 (but not the catalytically inactive H225A mutant) demonstrated USP30‐dependent stabilization of NCOA4, corroborated by CHX pulse‐chase assays and confocal colocalization studies.

Our findings revealed that USP30 deficiency alleviated H/R‐induced cell damage, consistent with the observations for METTL14. This alignment suggests that METTL14 may mediate USP30 methylation, thereby influencing its expression. To further confirm this possibility, fluorescence in situ hybridization (FISH) revealed nuclear colocalization of METTL14 and USP30. Subsequently, MeRIP and m6A immunoprecipitation sequencing identified m6A modification sites within USP30. To validate the role of METTL14 in stabilizing USP30 mRNA via YTHDF1, we overexpressed METTL14 and observed m6A modification in the 5' UTR of USP30 mRNA (as evidenced by MeRIP and truncation mutation experiments). METTL14 overexpression upregulated USP30 mRNA levels, an effect abolished by YTHDF1 silencing. Moreover, YTHDF1 suppressed the degradation of USP30 mRNA. These findings suggest that METTL14‐dependent m6A modification of USP30 facilitates YTHDF1 binding. In our experimental setting, YTHDF1 appears to promote USP30 expression at least in part by increasing USP30 mRNA stability, although an additional effect on translation cannot be excluded.

A limitation of the present study is that the human lung specimens were obtained from end‐stage ARDS patients undergoing lung transplantation and therefore reflect advanced‐stage disease rather than the early acute injury phase modeled in mice. Thus, the human data were used primarily to support the translational relevance of the METTL14/YTHDF1‐USP30‐NCOA4 axis in severe lung injury. In addition, although TC‐1 cells provided a practical murine epithelial system for mechanistic work, they do not capture the full cellular heterogeneity of the injured lung or the multicellular interactions present in vivo. Future studies incorporating spatial transcriptomics, multi‐omics approaches, and cell‐type‐specific models will help further define the cellular landscape of I/R‐induced lung injury. Because apoptosis was assessed mainly by TUNEL staining at a single time point, the present data do not exclude a context‐dependent contribution of apoptosis.

Our findings delineate a METTL14‐driven epitranscriptomic axis in which m6A modification of USP30 mRNA enhances transcript stability and translational efficiency through YTHDF1 recognition. This post‐transcriptional regulation elevates USP30 protein levels, which suppress NCOA4 ubiquitination and proteasomal degradation. Stabilized NCOA4 amplifies ferritinophagy, liberating intracellular Fe^2^^+^ to fuel mitochondrial lipid peroxidation and ferroptosis in lung epithelial cells. This cascade of events, spanning m6A‐dependent gene regulation and iron dyshomeostasis, collectively exacerbates ischemia‐reperfusion‐induced ALI, highlighting novel therapeutic targets for intervention (Graphical Abstract).

## 4 Materials and Methods

### 4.1 Clinical Study on Patients With ARDS

Lung tissues were obtained from three end‐stage ARDS patients (American‐European Consensus Conference criteria: PaO^2^/FiO^2^ ≤ 200, acute bilateral infiltrates) undergoing transplantation (2020–2022) and five age‐matched controls providing histologically normal tissues (> 2 cm from pulmonary nodule margins). The protocol (Ethics No. 2023138k, Zhongnan Hospital) included written informed consent.

### 4.2 Public scRNA‐seq Dataset Acquisition and Analysis

Publicly available scRNA‐seq data analyzed in this study were obtained from the Gene Expression Omnibus (GEO) under accession number GSE264278. The dataset was reanalyzed using Seurat (v4.3.0) for quality control, clustering, dimensionality reduction, visualization, and differential expression analysis. Gene Ontology (GO) enrichment analysis was performed using clusterProfiler (v4.0).

### 4.3 Histological Analysis

A semi‐quantitative injury score (0–4 per criterion: hemorrhage, edema, leukocyte infiltration) was assessed independently by two pathologists blinded to group allocation using a Nikon Eclipse E200 microscope. Discrepant scores were resolved by joint review.

### 4.4 Immunohistochemical Staining

After antigen retrieval (microwave heating), sections were incubated overnight at 4°C with rabbit anti‐METTL14 antibody (1:1000, Abcam, China, #ab309096). Species‐matched fluorescent/biotin‐labeled secondary antibodies were applied, and images were captured using a Nikon microscope.

### 4.5 IF Assays

Cells were probed with anti‐METTL14 (Abcam, China, #ab309096) and anti‐USP30 (Abcam, China, #ab314749) at 4°C overnight, followed by AlexaFluor‐488 secondary antibodies and DAPI counterstaining. Images were acquired using a Zeiss LSM 800 Airyscan. For fluorescence‐based image quantification, images were analyzed using ImageJ by investigators blinded to group assignment. Multiple randomly selected non‐overlapping fields per section or dish were quantified and averaged to generate one biological replicate. For Figure 6A, a combined IF–RNA FISH assay was performed in TC‐1 cells to visualize METTL14 protein and USP30 transcript localization. METTL14 was detected by IF, USP30 was detected by RNA fluorescence in situ hybridization, and nuclei were counterstained with DAPI.

### 4.6 Western Blot

Protein lysates from mouse lungs or cells were quantified using a BCA protein assay kit (Beyotime, China). After being separated by 10% SDS‐PAGE, protein samples (20 µg per group) were transferred to PVDF membranes. The membranes were blocked with 5% non‐fat milk and incubated overnight at 4°C with primary antibodies: β‐actin (1:20,000, Affinity, China, #T0022), NCOA4 (1:1000, Abcam, China, #ab314553), METTL14 (1:1000, Abcam, China, #ab309096), USP30 (1:1000, Abcam, China, #ab314749), YTHDF1 (1:1000, Affinity, China, #DF3422), FTH1 (1:1000, Affinity, China, #DF6278), and LC3 (1:2000, Abcam, China, #Ab192890). Membranes were washed (TBST) and incubated with HRP‐conjugated secondary antibodies (goat anti‐rabbit, 1:1000, Beyotime, China, #A0208; goat anti‐mouse, 1:10,000, Proteintech, China, #SA00001‐1). The bands were examined using ImageJ software after being detected using an ECL kit (Servicebio, China, #G2014).

### 4.7 Animals

Male C57BL/6 mice (8–10 weeks old, 22–25 g) were purchased from the Wuhan University Animal Experiment Center. Mice were assigned to experimental groups using a random‐number‐based allocation procedure. METTL14^fl/fl^‐Sftpc‐Cre conditional knockout mice were generated via CRISPR‐Cas9‐mediated gene editing (GemPharmatech Co., Ltd.). METTL14 lung epithelial cell conditional knockout mice were produced by crossing METTL14^flox^ mice with Sftpc‐Cre mice. Littermate METTL14^flox^ mice served as the control group. Mice were divided into four groups (*n* = 6) and subjected to I/R or sham surgery: METTL14^fl/fl^+Sham, METTL14^fl/fl^+I/R, METTL14‐cKO+Sham, and METTL14‐cKO+I/R groups. All procedures complied with NIH guidelines and were approved by the Animal Ethics Committee of Wuhan University (Protocol #ZN2025004).

### 4.8 Mouse Model of Ischemia/Reperfusion Injury

Mice were anesthetized with sodium pentobarbital (35 mg/kg, i.p.) and ventilated via Harvard‐Inspiratory ASVP ventilator (10  µl/g tidal volume, respiratory rate 120/min, 21% FiO_2_). Left lung ischemia was induced by hilar clamping (1 h), followed by 2 h reperfusion. In animals on single‐lung ventilation, the tidal volume was reduced to 6 µL/g, and the respiratory rate was increased to 150 breaths per minute. Sham controls underwent thoracotomy without clamping and matched ventilation duration. Heparin (500 U/kg, i.p.) was administered pre‐clamping. Terminal anesthesia (pentobarbital 100 mg/kg) was used for tissue harvest.

### 4.9 Quantification of Global m6A Levels

Global m6A levels in lung mRNA were analyzed via Agilent 6460 QQQ LC‐MS/MS (MRM mode). Poly(A)‐selected RNA (NEBNext E7490) was digested to nucleosides and separated on an Agilent 1260 HPLC with detection of *m*/*z* transitions: 268→136 (A) and 282→150 (m6A). Absolute quantification used nucleoside standard curves, with m6A levels expressed as m6A/A ratio.

### 4.10 Dot‐Blot Assay

RNA was isolated using Sigma mRNA Mini‐Prep Kit, spotted onto Hybond‐N+ membranes, and UV‐cross‐linked (120 mJ/cm^2^). Membranes were probed with anti‐m6A antibody (1:10,000, Synaptic Systems, China, #202003) and HRP‐conjugated IgG (1:5,000), with signals detected by ECL (Beyotime, China). RNA loading was normalized via methylene blue staining.

### 4.11 Quantitative RT‐PCR (qPCR)

Total RNA was extracted with TRIzol (Invitrogen, USA) and reverse‐transcribed into cDNA using PrimeScript RT Reagent Kit (Takara, China). TB GreenPremix Ex Taq II (Takara, China) was used for qPCR. Primer sequences are listed in Table S2. β‐Actin served as the endogenous control.

### 4.12 Determination of ROS

TC‐1 cells were stained with 10 µM dihydroethidium (DHE, Sigma D7008) or 5 µM BODIPY‐581/591‐C11 (Thermo Fisher) for intracellular/lipid ROS. Fluorescence was quantified via Nikon TE‐2000 microscope (485/530 nm) or BD Accuri C6 Plus flow cytometry. Lung cryosections (10 µm) were incubated with 50 µM DHE (37°C, 30 min) and imaged (525/610 nm). Data were analyzed with FlowJo v10.8.1.

### 4.13 TEM

Lung tissues were fixed in 2.5% glutaraldehyde (90 min) and 1% osmium tetroxide, stained with 2% uranyl acetate/lead citrate, and embedded in epoxy resin. Ultrathin sections (100 nm) were imaged on an HT‐7500 TEM (Hitachi). Mitochondrial damage (vacuolization, cristae loss) was quantified in 10 random fields per section across three sections per group [40].

### 4.14. MeRIP‐seq and RNA‐seq

4.14

RNA from I/R and control lungs underwent m6A immunoprecipitation (MeRIP) with anti‐m6A antibody (1:10,000, Synaptic Systems, China, #202003), followed by library preparation using the KAPA Stranded mRNA‐Seq Kit and 150‐cycle paired‐end sequencing (Illumina HiSeq 4000). Reads were aligned to GRCm38 with HISAT2 (v2.1.0). Differentially expressed genes were identified via Ballgown, while ExomePeak analyzed m6A peaks using MeRIP/input data.

### 4.15 Three‐Dimensional Structure Prediction

Human NCOA4 (UniProt ID: Q13772) and USP30 (UniProt ID: Q70CQ3) sequences were retrieved from UniProt. Full‐length structural models were generated using AlphaFold3 to estimate a putative binding mode between USP30 and NCOA4. Binding energy (PRODIGY‐calculated ΔG = −14.6 kcal/mol) and candidate contact regions were then analyzed by hydrogen‐bond and hydrophobic interaction mapping.

### 4.16 Cell Culture and Hypoxia‐Reoxygenation (H/R) Model

TC‐1 cells were used as a tractable murine epithelial model because they tolerate H/R treatment well and allow efficient genetic manipulation for mechanistic studies of ferroptosis‐related signaling. Cells were maintained in DMEM supplemented with 10% FBS. H/R injury was induced by 24 h hypoxia (1% O_2_) followed by 2 h reoxygenation (21% O_2_) [31]. Prior to hypoxia exposure, cells were pretreated with lentiviral constructs (METTL14‐OE, USP30‐OE, YTHDF1‐OE) or siRNA (si‐METTL14, si‐USP30; Sangon, Wuhan, China). TC‐1 cells were randomly distributed into si‐NC+Con, si‐NC+H/R, si‐METTL14+Con, and si‐METTL14+H/R groups. Lentiviruses (GeneChem) and the pGL3 luciferase reporter plasmid (Genechem) were transfected per manufacturer protocols.

### 4.17 Co‐IP Assay

TC‐1 lysates in RIPA buffer (1% NP‐40, protease/phosphatase inhibitors) were pre‐cleared with protein A/G agarose beads (2 µg IgG, 1 h). Supernatants were incubated with anti‐NCOA4 (1:1000, Abcam, China, #ab314553, 24 h). Immune complexes were captured with A/G beads (2 h, 4°C) and eluted in SDS‐PAGE buffer (95°C) for western blotting.

### 4.18 Cell Transfection

TC‐1 cells were transduced with lentiviral particles encoding METTL14, USP30, or YTHDF1 to generate stable overexpression cell lines designated METTL14‐OE, USP30‐OE, and YTHDF1‐OE, respectively. For siRNA knockdown, TC‐1 cells were transfected with siRNAs targeting METTL14, USP30, or YTHDF1 (Shanghai GenePharma, China) using Lipofectamine 2000 (Thermo Fisher Scientific, USA), with siNC as a negative control. Transfected cells were selected 48 h post‐transfection for downstream assays. TC‐1 cells were randomly distributed into Con, Vector, METTL14, si‐NC, and si‐METTL14 groups.

### 4.19 MeRIP qPCR

The m6A immunoprecipitation (MeRIP) qPCR was performed following a published procedure [41]. Total RNA from mouse lungs or TC‐1 cells was fragmented, and m6A‐modified transcripts were immunoprecipitated (IP) with anti‐m6A antibody‐bound beads supplemented with RNase inhibitor at 4°C overnight. A portion of the RNA sample was stored as input control. RNA from IP and input samples was reverse‐transcribed, and m6A enrichment was quantified via qRT‐PCR (SYBR Green, Applied Biosystems) using gene‐specific primers.

### 4.20 Dual‐Luciferase Reporter Assay

TC‐1 cells were co‐transfected with the pGL3 luciferase vector and Renilla control plasmid (Promega, USA). After 48 h, lysates were analyzed using the Dual‐Luciferase Reporter Assay System (Promega) on a SpectraMax M5 microplate reader (Molecular Devices, USA). Firefly luciferase activity was normalized to Renilla.

### 4.21 Detection of Iron in Mitochondria

Mitochondrial Fe^2^^+^ levels were assessed using the fluorescent probe Mito‐FerroGreen (Dojindo, Japan). Following H/R, TC‐1 cells were washed thrice with serum‐free media and incubated with 5 µmol/L ferrocyanide (5 µmol/L) at 37°C for 30 min. Cells were rinsed with PBS, and fluorescence intensity was quantified using a Nikon TE‐2000 fluorescence microscope (Tokyo, Japan) at Ex/Em = 488/530 nm.

### 4.22 Statistical Analysis

Quantitative data are presented as mean ± SD unless otherwise stated. Clinical variables with non‐normally distributed data are presented as median (interquartile range). For comparisons between two groups, an unpaired two‐tailed Student's *t*‐test was used for normally distributed data, whereas the Mann–Whitney *U* test was used for non‐normally distributed data. For comparisons among three or more independent groups, including sham and multiple reperfusion‐time groups, one‐way ANOVA followed by Tukey's post hoc test was used. Categorical variables were analyzed using Fisher's exact test or the chi‐square test, as appropriate. Statistical analyses were performed using GraphPad Prism 8.0. In human experiments, *n* indicates the number of patients or tissue samples; in animal experiments, *n* indicates the number of mice per group; and in cell experiments, *n* indicates the number of independent biological replicates, unless otherwise specified. A *p* value < 0.05 was considered statistically significant.

## Author Contributions

Aming Sang, Qiongyue Zhang, and Xianwu Zhou contributed equally to this work. Aming Sang, Qiongyue Zhang, Xianwu Zhou, Li Li, Ling Li, and Xinyi Li contributed to the conception and design of the study. Aming Sang, Qiongyue Zhang, Xianwu Zhou, Xiaohua Wang, and Jin Wang performed the experiments and acquired the data. Xuemin Song provided guidance for manuscript revision and supervised additional experiments during revision. Xiaohua Wang and Jin Wang contributed to methodology development and technical support. Aming Sang, Qiongyue Zhang, and Xianwu Zhou analyzed and interpreted the data. Aming Sang drafted the manuscript. Qiongyue Zhang, Xianwu Zhou, Xiaohua Wang, Jin Wang, Xuemin Song, Li Li, Ling Li, and Xinyi Li revised the manuscript critically for important intellectual content. Li Li, Ling Li, and Xinyi Li obtained funding, supervised the study, and provided administrative and scientific oversight. All authors read and approved the final manuscript.

## Funding

This study was supported by the National Natural Science Foundation of China (No. 82472204), Translational Medicine and Interdisciplinary Research Joint Fund of Zhongnan Hospital of Wuhan University (Grant No. ZNJC202303), Clinical Medicine Plus X‐Outstanding Young Scholars Project, Wuhan University, and the Fundamental Research Funds for the Central Universities (No. 2042025YXB019).

## Ethics Statement

All animal experiments were approved by the Wuhan University's Animal Ethics Committee (Protocol #ZN2025004) and conducted in compliance with the NIH Guide for the Care and Use of Laboratory Animals. Human lung tissue samples were obtained from end‐stage ARDS patients undergoing lung transplantation at Zhongnan Hospital, with approval from the Zhongnan Hospital Institutional Review Board (Protocol No. 2023138k). Written informed consent was obtained from all participants or their legal representatives prior to sample collection. Individual patient data are fully anonymized, and no identifiable images or personal details are included in this study.

## Conflicts of Interest

The authors declare no conflicts of interest.

## Supporting information




**Supporting File 1**: mco270959‐sup‐0001‐SuppMat.docx

## Data Availability

The scRNA‐seq dataset analyzed in this study was obtained from the public GEO repository under accession number GSE264278. All other data supporting the findings of this study are included in the article and its Supporting Information or are available from the corresponding author upon reasonable request.
