# Supplementary material for: METTL14‐Mediated N6‐Methyladenosine Exacerbates Acute Lung Injury via USP30/NCOA4‐Dependent Ferroptosis
Source: MedComm (2020). 2026 Aug 28;7(9):e70959. doi: 10.1002/mco2.70959 (PMC13522826; doi:10.1002/mco2.70959)
Supplement: Supplementary file 1 [file MCO2-7-e70959-s001.docx]

**METTL14-mediated N6-methyladenosine exacerbates acute lung injury via USP30/NCOA4-dependent ferroptosis**

Aming Sang ^1, 5, #^, Qiongyue Zhang ^1, 5, #^, Xianwu Zhou ^1, 5, #^, Xiaohua Wang ^1^, Jin Wang ^1^, Xuemin Song ^1^，Li Li ^2,3, *^, Ling Li ^6, *^, Xinyi Li ^1, 5, *^

1 Department of Anesthesiology, Zhongnan Hospital of Wuhan University, Wuhan, Hubei, China.430071.

2 Department of Critical Care Medicine, Zhongnan Hospital of Wuhan University, Wuhan, China.430071.

3 Clinical Research Center of Hubei Critical Care Medicine, Wuhan, Hubei, China.430071.

4 Department of Cardiovascular Surgery, Zhongnan Hospital of Wuhan University，Wuhan, Hubei, China.430071.

5 Hubei Provincial Engineering Research Center of Minimally Invasive Cardiovascular Surgery, Wuhan, Hubei, China.430071.

6 Zhongnan Hospital of Wuhan University, Institute of Hepatobiliary Diseases of Wuhan University, Transplant Center of Wuhan University, National Quality Control Center for Donated Organ Procurement, Hubei Key Laboratory of Medical Technology on Transplantation，Hubei Provincial Clinical Research Center for Natural Polymer Biological Liver, Wuhan Hubei，430071.

#These authors contributed equally to this work.

*Correspondence:

Xinyi Li, [lxy08272021@163.com](mailto:lxy08272021@163.com)

Ling Li, [zn000426@whu.edu.cn](mailto:zn000426@whu.edu.cn)

Li Li, [mrcatking@gmail.com](mailto:mrcatking@gmail.com)

Figure S1. Validation of METTL14, USP30, and NCOA4 expression in human end-stage ARDS lung tissues. **(A)** H&E staining of control and end-stage ARDS lung tissues. **(B)** IHC staining in lung tissue. **(C)** Immunofluorescence staining in lung tissue. **(D-E)** Western blot analysis was performed to quantify the protein expression levels of NCOA4, USP30, and METTL14 in lung tissue. (three patients with end-stage ARDS constitute the experimental group, and five patients undergoing pulmonary benign nodule resection serve as the control group). (F-G) CHX chase analysis of NCOA4 protein stability in TC-1 cells expressing control shRNA or shRNA-METTL14. Data are shown as the mean ± SD. **P* < 0.05, ***P* < 0.01,****P* < 0.001 for indicated comparisons.

Figure S2. (A) Images of apoptosis in lung tissue stained by green fluorescence. (B) The representative immunofluorescent staining shows the image of 4-HNE. (C) Quantitative results of apoptosis rate. (D) the quantification of 4-HNE immunofluorescence intensity. (E) qPCR for measuring FTH1 mRNA levels. (*n* = 6). Data are shown as the mean ± SD. **P* < 0.05, ***P* < 0.01,****P* < 0.001 for indicated comparisons.

Figure S3. The three-dimensional structure of NCOA4 (UniProt ID：Q13772) bound to USP30 (UniProt ID：Q70CQ3) was predicted using AlphaFold3. (A) The optimal binding pose is illustrated in cartoon representation. (B) The protein-protein binding process was visualized through molecular surface representation. (C) The green cartoon representation depicts protein NCOA4, while the blue cartoon corresponds to protein USP30. Molecular interactions are visualized as follows: gray dashed lines for hydrophobic interactions, blue solid lines for hydrogen bonds, green dashed lines for π-π stacking, orange dashed lines for π-cation interactions, yellow dashed lines for salt bridges, and yellow spheres indicating valence electron centers.

Figure S4. (A) Images of intracellular ROS levels in TC-1 cells stained by DCFH-DA (10 μM) fluorescent probes (Bar = 50 μm). (B) Quantitative results of ROS. (C) Cell viability using CCK-8 kit. (n = 6). Data are shown as the mean ± SD. **P* < 0.05, ***P* < 0.01,****P* < 0.001 for indicated comparisons.

Table S1. Parameter Values (expressed as mean ± SEM unless otherwise specified)

| **Variables** | **Normal range** | **Control group** | **End-stage ARDS group** | ***P* Value** |
| --- | --- | --- | --- | --- |
| **Complete blood count** | | | | |
| Platelet count (×10⁹/L) | 125-350 | 172.50±105.57 | 118.38±78.92 | 0.009 |
| ^#^Neutrophil count (×10⁹/L) | 1.8-6.3 | 4.76 (3.65-7.34) | 12.21 (8.99-16.89) | 0.007 |
| Monocyte (%) | 3-10 | 6.72±3.19 | 3.42±2.35 | 0.002 |
| White blood cell count (×10⁹/L) | 3.5-9.5 | 6.35±3.68 | 15.65±8.32 | 0.012 |
| Hemoglobin (g/L) | 130-175 | 119.67±31.49 | 108.37±28.58 | 0.054 |
| ^#^Basophil count (×10⁹/L) | 0-0.06 | 0.02 (0.01-0.03) | 0.03 (0.02-0.07) | 0.265 |
| ^#^Lymphocyte count (×10⁹/L) | 1.1-3.2 | 0.79 (0.40-1.31) | 0.46 (0.36-0.86) | 0.488 |
| Monocyte count (×10⁹/L) | 0.1-0.6 | 0.54±0.26 | 0.48±0.36 | 0.776 |
| ^#^Eosinophil count (×10⁹/L) | 0.02-0.2 | 0.006 (0-0.02) | 0.014 (0-0.08) | 0.754 |
| **Biochemical test** | | | | |
| Albumin (g/L) | 40-55 | 35.29±7.80 | 26.78±4.54 | 0.001 |
| ^#^Blood urine nitrogen (mmol/L) | 2.8-7.6 | 9.37 (6.78-14.51) | 11.97 (9.32-23.50) | 0.275 |
| ^#^Brain natriuretic peptide (pg/mL) | <100 | 149.20(60.85-330.45) | 220.30(65.60-850.40) | 0.977 |
| ^#^Potassium (mmol/L) | 3.5-5.3 | 4.21 (3.92-4.60) | 4.98 (4.25-5.79) | 0.048 |
| Total plasma protein (g/L) | 65-85 | 62.83±7.13 | 57.18±8.40 | 0.013 |
| ^#^Aspartate aminotransferase (U/L) | 15-40 | 49.00 (25.00-65.00) | 63.00 (31.00-245.00) | 0.488 |
| ^#^Creatinine (µmol/L) | 64-104 | 89.35 (77.80-124.80) | 121.50 (70.00-325.60) | 0.273 |
| Uric acid (µmol/L) | 208-428 | 388.08±108.97 | 365.74±189.04 | 0.575 |
| ^#^Alanine aminotransferase (U/L) | 9-50 | 38.00 (15.00-47.00) | 42.50 (13.00-73.00) | 0.489 |
| ^#^Globulin (g/L) | 20-30 | 30.50 (28.30-32.10) | 29.90 (24.80-32.70) | 0.268 |
| Sodium (mmol/L) | 137-147 | 141.09±10.81 | 137.82±7.97 | 0.312 |
| ^#^Lactate dehydrogenase (U/L) | 125-243 | 470 (418-650) | 565 (440-660) | 0.990 |
| ^#^Creatinine kinase-MB (U/L) | <171 | 33 (21-45) | 78 (54-148) | 0.632 |
| ^#^Highly sensitive troponin I (pg/mL) | 0-26.2 | 22.1 (11.8-116.0) | 68.5 (26.0-206.8) | 0.287 |
| **Inflammatory profile** | | | | |
| ^#^C-reactive protein (mg/L) | 0-10 | 88.90 (28.36-162.55) | 176.70 (134.10-320.40) | 0.295 |
| ^#^Procalcitonin (ng/mL) | < 0.05 | 0.66 (0.12-2.78) | 4.68 (1.52-11.58) | 0.030 |
| **Coagulation profile** | | | | |
| ^#^Activated partial  thromboplastin time (s) | 21.5-36.5 | 28.65 (26.30-31.70) | 31.95 (29.30-38.40) | 0.059 |
| Fibrinogen (mg/dL) | 238-498 | 410.94±108.16 | 424.11±155.36 | 0.675 |
| ^#^D-dimer (mg/L) | 0-500 | 502.50(278.00-2159.00) | 3512.50(2767.00-10899.00) | 0.002 |
| ^#^Prothrombin time (s) | 9.4-12.5 | 12.30 (11.60-13.50) | 14.65 (12.70-16.60) | 0.023 |
| **Blood gas analysis** | | | | |
| SaO₂ | 0.95-0.99 | 0.92±0.08 | 0.70±0.27 | 0.040 |
| PH | 7.35-7.45 | 7.42±0.08 | 7.06±0.21 | < 0.001 |
| PO₂ (mmHg) | 83-108 | 72.1±32.04 | 60.53±23.34 | 0.371 |
| PCO₂ (mmHg) | 35-45 | 33.64±11.14 | 61.53±19.49 | 0.001 |
| BE (mmol/L) | -2.3 - +3 | -3.84±6.14 | -11.67±10.80 | 0.088 |
| HCO3- (mmol/L) | 21.4-27.3 | 19.82±6.15 | 17.77±8.23 | 0.483 |
| **Bilateral involvement in chest**  **radiographs-n** (%) | NA | 4 (80) | 3 (100) | -- |

^#^ The data of abnormal distribution is expressed as median and IQR.

Table S2: Primer sequences used in the study

| **GENE** | **Primer (5′ → 3′)** |
| --- | --- |
| USP30 | Forward primer: GCGGCCGTCAGATACAAAGT |
|  | Reverse primer: TGGCAGGACAGAGCTTTCAG |
| METTL14 | Forward primer: CTCCAGGTCGGAGTGTGAAC |
|  | Reverse primer: AACCGTTTAAACCAGCCCCT |
| YTHDF1 | Forward primer: CTGCAGTTAAGACGGTGGGT |
|  | Reverse primer: TAGCAATGGCTGCCCATGAA |
| NCOA4 | Forward primer: GGGCAACCTCAGCCAGTTAT |
|  | Reverse primer: GGGATCTGAAAATTCCCAACGG |
| USP11 | Forward primer: AATTAGCCTGCCAGGAGAGG |
|  | Reverse primer: CTCAGCAAGCTCACCCTTCA |
| USP14 | Forward primer: GCCGGTTTGAATGAGGCTTG |
|  | Reverse primer: CAGCCGAGGAATACGAAGCA |
| OTUD3 | Forward primer: TAAGGCCATGTCCCGAAAGC |
|  | Reverse primer: TCCCTTGGTTCATCCGAAGC |
| β-actin | Forward primer: CACTGTCGAGTCGCGTCC |
|  | Reverse primer: TCATCCATGGCGAACTGGTG |
